# The first 30 days of COVID-19 vaccination in Cameroon: achievements, challenges, and lessons learned

**DOI:** 10.11604/pamj.2022.41.201.30218

**Published:** 2022-03-14

**Authors:** Adidja Amani, Dove Djossaya, Andreas Ateke Njoh, Andre Arsene Bita Fouda, Shalom Ndoula, Haamit Mahammat Abba-kabir, Tatiana Mossus, Georges Nguefack-Tsague, Joseph Kamgno

**Affiliations:** 1 Sub-directorate of Vaccination, Directorate of Family Health, Cameroon Ministry of Public Health, Yaoundé, Cameroon,; 2 Faculty of Medicine and Biomedical Sciences, University of Yaoundé I, Yaoundé, Cameroon,; 3 Health Research Econometrics National Institute of Statistics, Yaoundé, Cameroon,; 4 Expanded Program on Immunization, Yaoundé, Cameroon,; 5 Euclid University, Bangui, Central African Republic,; 6 World Health Organization Afro Communicable and Non-Communicable Diseases/Vaccine Preventable Diseases

**Keywords:** COVID-19, vaccines, achievements, challenges, lessons learned, Cameroon

## Abstract

**Introduction:**

Cameroon's national vaccination campaign was launched on April 12, 2021, amid a nationwide outbreak of COVID-19 with two types of vaccines. This study provides preliminary evidence to assess early outputs of the COVID-19 vaccination response strategy implementation.

**Methods:**

a cross-sectional study was conducted from April 12, 2021, to May 11, 2021, and data on COVID-19 vaccination were reviewed from the Ministry of Public Health database. Descriptive statistical analyses were conducted.

**Results:**

thirty days after the introduction of COVID-19 vaccines, just about five percent of the target population was vaccinated. Women represented one-third of the people vaccinated regardless of age and health conditions. Although AEFI reported were minor and scanty with both vaccines, most of the vaccinated did not come back for their second dose. There was a need to build confidence among eligible beneficiaries to expand the benefits of vaccination to control the current pandemic.

**Conclusion:**

the country was still far below the target, which was worrisome given that vaccine uptake was slow. Also, 391 200 doses of the Covishield were at risk of expiration in August 2021. This study offers insights into those early efforts contributing to significant discussions about the approaches to improve service delivery and vaccine uptake.

## Introduction

Since the detection of the first positive case of COVID-19 in Cameroon on March 6, 2021, there was an upsurge in cases across the country. According to the 73^rd^ situation report, 57,337 confirmed cases of COVID-19 were recorded with 243 health workers affected and 851 deaths, i.e. a case fatality rate of 1.5%. Therefore, Cameroon was in its second wave, which was more deadly than the first. The resurgence of COVID-19 cases and the occurrence of a new wave of contaminations in Cameroon, between February 25 and March 3, 2021 [1], arose when the scientific community had a new response to this pandemic. The COVID-19 vaccination was being introduced in many countries.

As part of the response to the COVID-19 pandemic, Cameroon subscribed to the COVAX facility launched in April 2020. The COVAX facility is the vaccine pillar accelerator of Access to COVID-19 Tools (ACT), aiming for innovative and equitable access to diagnosis, treatment, and vaccine [2]. It is a global mechanism for pooling resources and requests for COVID-19 vaccines to ensure that low-income countries have access to COVID-19 vaccines at the same time as high-income countries. To this end, Cameroon has developed a National Deployment and Vaccination Plan whose initial vaccination approach provided for a gradual deployment of vaccines through 15 pilot sites identified for the first vaccine allocation [2].

On Sunday April 11, 2021, the Prime Minister of Cameroon received a donation of 200,000 doses of Sinopharm from the Chinese Government. As the country was eligible for the advance market commitment 92 financing mechanism of the COVAX facility [3], on April 17, the country received 391,200 doses out of the 1,200,000 doses awaited of Covishield vaccines. In order to reduce morbidity and mortality, the country also opted for a vaccination campaign with Sinopharm and Covishield vaccines as primary targets: healthcare workers, security personnel, people over 50 years of age, and people with comorbidities. The said campaign took place from April 26 to 30, 2021, in the country's ten regions. The organization of the COVID-19 response campaign by the Government was motivated by the following considerations: 1) the epidemiological situation of the country with an increased number of cases and deaths with community transmission; 2) the reduction in morbidity and mortality due to COVID-19; 3) to maintain health services; 4) to ensure the reduction of transmission to decrease socio-economic disruption. The country aimed to vaccinate 5,400,000 people against COVID-19 by the end of 2021, then 15 million Cameroonians by December 2022 to reach the threshold vaccination coverage expected to confer herd immunity [4].

With more than 80,000 cases of COVID-19 confirmed as of June 11 and a case fatality rate of 1.6%, Cameroon intensified and expanded the target of the vaccination campaign from June 7 to 11 to all persons over the age of 18, including in the organized group made up of industries, public administration, and prisons. The Minister of Public Health of Cameroon launched the deployment of COVID-19 vaccination on April 12 at the specialized center for COVID-19 patients of the Central hospital in the capital city of Yaoundé. Following the implementation of any new vaccination program, an evaluation of its coverage, effectiveness, and safety in a real-life setting is essential as the first days of a new vaccination program are crucial to establishing the routines. This paper intends to assess early outputs of implementing the COVID-19 vaccination response strategy in terms of vaccine uptake, coverage, AEFI surveillance, vaccination timeliness of the second dose and communication.

## Methods

This cross-sectional study was conducted over four weeks, between April 12, 2021, and May 11, 2021, in Cameroon upon COVID-19 vaccine introduction. The campaign's goal was to reduce morbidity and mortality from COVID-19 by vaccinating those most at risk with one of the following vaccines: Sinopharm and Covishield. The campaign objectives were: i) to vaccinate 70% of health workers and people aged over 50 with comorbidities; ii) ensure adequate information of at least 90% of the population of all heath districts; iii) to actively investigate and report any case of AEFI; iv) to ensure effective vaccine management. Cognisant that the mode of administration of these two vaccines was by injection, the vaccines were administered only by previously trained health personnel. Thus, the main vaccination strategies during this campaign were twofold: fixed vaccination in all identified vaccination centers, and mobile vaccination strategy in public places, care centers for the elderly and people with comorbidities, places of worship, public and private services, markets, penitentiary centers, chiefdoms, health facilities.

**Setting:** the Republic of Cameroon is a country in Central Africa, stretching from the Gulf of Guinea to Lake Chad. Its particular geographical location explains the diversity of its climate and its natural landscape. Cameroon has an area of 475,650 km^2^. It is limited to the east by the Republic of Central Africa, in the North-East by the Republic of Chad, in the South by the Republic of Congo, the Republic of Gabon and the Republic of Equatorial Guinea, in the southwest by the Atlantic Ocean and west by the Federal Republic of Nigeria. Based on demographic projections [5], the population of Cameroon is estimated at approximately 27,142,557 inhabitants with a median age of 18.7 years. The introduction of vaccination against COVID-19 in the form of a campaign followed many steps: i) accreditation of vaccination centers that possessed appropriate cold chain and sufficient human resources; ii) establishment of mobile teams trained and equipped with adequate cold chain equipment; iii) training of all health personnel in screening, treatment of COVID-19 and administration of vaccines; iv) strengthening of the pharmacovigilance system to monitor AEFI; v) mobilization of financial resources for genomic surveillance. Vaccination centers were identified in each health district following three criteria: i) the availability of qualified and trained personnel in vaccine management and immunization service delivery; it could be a doctor and/or a pharmacist, and at least 2 nurses; ii) The availability of a reliable cold chain equipment; iii) sufficient storage capacity for vaccines. Selected vaccination centers were to organize advanced strategies to reach the entire target in their coverage area, including institutions, associations for the elderly, prisons, refugee camps, or settlements for internally displaced persons. The first round of the campaign took place from April 26 to 30, 2021, in the 10 regions. Vaccination was carried out in strict compliance with measures to limit the spread of COVID-19. Vaccination sites were set up for this purpose while respecting physical distancing, screening of people to be vaccinated and the use of personal protective equipment during vaccination according to the guidelines on the continuity of immunization in the context of COVID-19.

**Participants:** the National Immunization Technical Advisory Group and the Scientific Committee for COVID-19 responses prioritized the target populations for the vaccination against COVID-19 as follows: front-line health and social workers, people over 50 years, persons who are critical for the running of the State; refugees over 50, staff from Embassies and diplomatic missions accredited to Cameroon, people under the age of 50 with comorbidities with a significant higher risk of severe illness or death and eligible refugees under the age of 50 years old. In addition, workers under 50 years old critical functioning of the state including government, administrative bodies, parliamentarians, judiciary, regional councilors, municipal councilors, eligible teachers, and students not taken into account in the previous group; and finally other target groups like travelers, transporters, prisoners, refugees, and other essential social sectors [2]. The vaccination campaign aimed at implementing the first scenario of National Deployment Vaccination Plan [NVDP] scenario, which targeted healthcare staff, security staff, people over 50, and people with comorbidities Table 1. A total of 244 vaccination centers across 190 districts were identified. In addition, 190 district supervisors, 244 fixed teams, 1,255 mobile teams 5,263 social mobilizers, and WHO and Unicef supervisors were deployed to implement this campaign. Moreover, 33 central supervisors which include 13 technical supervisors, ten communication supervisors, ten logistics supervisors, ten central coordinators, and ten data managers. At the regional level 212 actors were recruited which included ten for the coordination at the level of the governor's services, 20 people for regional coordination, 92 regional supervisors, ten pharmacovigilance focal points, 20 data managers, 20 logisticians, 20 accounting managers, and 20 communication focal points.

**Table 1:** estimate of the target population with scenario 1: the first vaccine acquisition intended for a target representing 3% of the total population 812,301

Scenario	Target groups	Estimate	Percentage of the total Population	% compared to the entire target to be vaccinated
The first vaccine acquisition for a target representing 3% of the total population 812,301	Primary health workers, including community health workers	72,558	0.3%	1%
	Security personnel	81,808	0.3%	2%
	People with comorbidities or conditions deemed to be at significantly higher risk of severe illness or death	258,401	1.0%	5%
	Elderly 50 and over (1/5 of this target)	399,893	1.5%	7%
**Total scenario 1**		812,660	3%	15%

**Variables:** the variables collected include: age, gender, health areas, population, vaccination start date, health workers status, doses administered, the first dose, the second dose, Adverse Event Following Immunization (AEFI), vaccine management, health condition, date of administration, vaccine type, vaccines lot number. Pending the establishment of the electronic data management system, all data were recorded on paper with a vaccination register, a stock management register, a vaccination card, population-based immunizations registers, and AEFI notification and investigation forms. A paper based data entry form summarized all the data at the district level with a gradual compilation up to the central level. These data were updated every day and shared with all partners after a monitoring meeting on Tuesdays at 10 a.m. The AEFI case notification data were transmitted through an Open Data Kit (ODK) form accessible on a server for the regional and central levels. These tools made it possible to follow the coverage, identify people vaccinated, keep the vaccination history, manage vaccines, and disseminate information. No individual-level data were available or included in the data files analyzed within the scope of this work. Vaccinated individuals were defined as people who received any of the two types of vaccines available within the period, from April 12, 2021, to May 12, 2021.

**Data availability:** COVID-19 vaccine administration data were obtained from the EPI database at the Cameroon Ministry of Public Health. Daily data on individual COVID-19 vaccination were compiled from the vaccination record of the 244 vaccination sites. Although the data management system gradually became automated, the vaccination register at the operational level collected contact details, socio-demographic and clinical information of the vaccinated person [sex, age, occupation, risk profile, etc].

**Data collection and management processes of the campaign:** initially, the campaign data collection and analysis tools were developed on Microsoft Excel and made available to the ten regions, 190 health districts, and 244 vaccination centers. Later on, the data entry mask was integrated into the District Health Information Software (DHIS2) to facilitate the daily vaccination data reporting. Thus data monitoring was done daily through the DHIS2 at all levels. The data completeness and quality were reviewed at different levels during daily coordination meetings.

**Implementation of the campaign:** the vaccination campaign was organized with Sinopharm and Covishield vaccines in the ten regions. This campaign ran from April 24, 2021, and targeted individuals over 18 years old. The objectives of the campaign were: administering to at least 200,000 people aged 18 and over, a dose of Covishield or Sinopharm vaccines, actively search and notify any case of AEFI, ensure effective vaccine management, and ensure the reporting of all vaccination data through the DHIS2.

**Before the campaign:** the training of central facilitators was organized at the central level before the start of the campaign. This training was cascaded down to the operational levels. In addition, the daily level of preparedness was monitored at all levels using an online dashboard; the monitored indicators included: availability of funds, quality of cold chain equipment, availability of consumables, and the daily supervision plan. The operational cost of this vaccination campaign was mainly covered by the government proper funds. However, additional funds were mobilized from partners including WHO, Unicef, and CHAI to support the implementation of communication activities and to deploy other actors in the field during the campaign.

**During the campaign:** during the campaign, the governor of each of the ten regions made an official launch of vaccination activities. At each region some central-level actors supported the coordination of activities. The central level and regional supervisors actors supported communication, vaccine management, reporting, and monitoring of AEFI in all districts. Daily evaluation meetings were carried out at each level. Daily coordination meetings were held at each level of the health pyramid [central, regional, and district] to monitor the communication activities, vaccine use, social mobilization, and vaccination data. Several strategies were implemented to ensure that a significant proportion of the population was reached. These included interpersonal communication, vaccination, the use of influential actors to communicate in their respective communities, and social media and journalists to spread sensitization messages. In order to strengthen the AEFI surveillance system, there was a cascade training of all AEFI focal points in the regions and districts before the start of vaccination. After the training, the actors used ODK in reporting all cases of AEFI. In addition, 573 vaccination teams were trained and equipped with AEFI management kits. Severe cases were investigated, and samples were collected and analyzed at the Centre Pasteur Laboratory of Yaoundé; afterwards the national AEFI review committee classified the reported cases.

**Ethical considerations:** the COVID-19 vaccination campaign and the data collection were organized following a ministerial decision to ensure the entire population is protected against COVID-19. The activities of vaccination received the authorization of the Ministry of Public Health of Cameroon N° D N°24/NS/MINSANTE/SG/DSF/GTC-PEV/SPA/PSV of 19 April 2021. Likewise, this research did not need consent for participation as it used aggregated secondary data from the ministry of health database.

## Results

**Figure 1:**
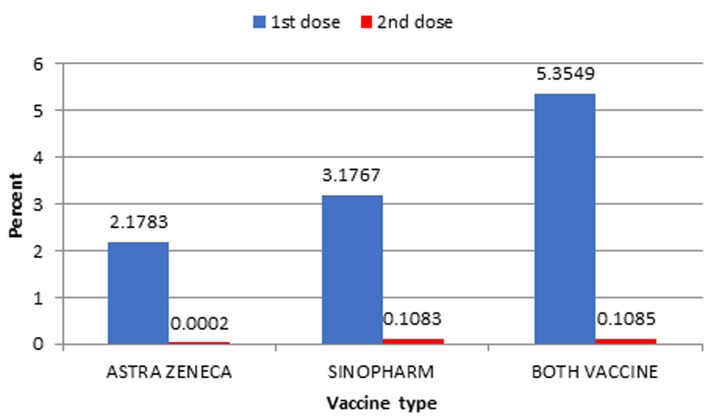
coverage (in percentage) by dose of each vaccine from April 12 to May 11 2021 (below vaccine uptake and coverage)

**Vaccine uptake and coverage:** from April 12 to May 12, 2021, 43,651 people were vaccinated with at least one dose. The 43,651 people have received their first dose and 1,061 have already received their second dose of vaccine against COVID-19 which represent 5.4% and less than 1% respectively of the target population. The First dose for Covishield stands at 2.18% against 3.18% for Sinopharm Figure 1.

**Progress in implementation:** there was a progressive rollout from 177 (93%) districts from the first week. By the end of the first thirty days, the new plan for deployment and vaccination was accelerated to ensure optimal uptake across the country and at this time, all 190 (100%) districts at the end of the first month were covered or rollout.

**Distribution by priority groups and sex:** health personnel represented 30 percent of the vaccinated population, and less than 12 percent of the vaccinated group presented comorbidities. Older adults age 50 years and women represented about 31 and 33 percent, respectively.

**Figure 2:**
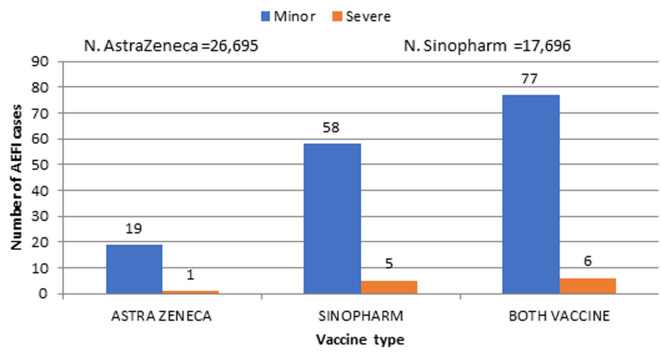
number of AEFI cases by vaccine type and form (below vaccine safety)

**Vaccine safety:** there was an active notification of AEFI. One death following a severe AEFI in the Adamawa region. The highest incidences of severe AEFI were in the South West and South regions with 0.54 and 0.32 per 1000 administered vaccine doses, respectively. In total, seventy-seven (0,017 per 1000 doses) minor cases and six severe cases of AEFI were reported during the first thirty days. More specifically, for Covishield, out of 26,695 doses administered, 19 (0.7 per 1000) minor cases and 1(0,04 per 1000) severe AEFI cases were reported as against 58 (3 per 1000) and 5 (0,3 per 1000) cases, respectively for Sinopharm out of a total of 17,696 doses administered Figure 2. Among the side effects reported, the most frequent were headache, fever and myalgia. The reported AEFI were further investigated and causality with the vaccines was established or ruled out. But this aspect is beyond the scope of this paper and would be presented in a further publication.

**Vaccination timeliness of the second dose:** the percentage of people who received at least one dose of a COVID-19 vaccine but did not receive a second dose is high (Figure 1). This percentage has an increasing trend, varying from 57.6% for April 12 to May 3, 2021 to 96.4% for April 20 to May 11 2021 Figure 3.

**Figure 3:**
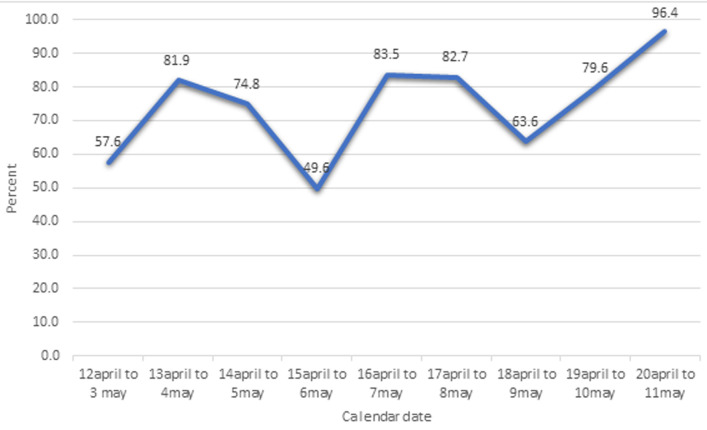
percentage of persons who received first dose COVID-19 vaccine but did not come for the second dose after 21 days (below vaccination timeliness of the second dose)

**Communication results:** the reporting of communication data was not systematic in all regions. With a total of 3,341 social mobilizers deployed in the field, 473,538 households were visited, 1,004,699 people were sensitized, and 670,695 targets were counted.

**Challenges:** despite the various efforts made at all levels, vaccination coverage is still very low. Three key challenges emerged from the actual experience in the first weeks of the COVID-19 vaccination namely logistics, financing, and communication. The difficulties encountered since the start of vaccination have been the adhesion and commitment of the community to vaccination, the presence of groups resistant to vaccination, non-compliance with barrier measures by certain actors, rumors surrounding the vaccine. Other challenges included low data reporting in DHIS2, rainy season making roads impassable, isolation of some localities, insecurity in some districts, with a health district named, Wabane losing 430 doses during a fire incident. The telephone network was absent in some areas, and challenges of data entry in ODK due to a poor configuration of the tool.

**The risk of great number of vaccines expiring:** one of the greatest challenges faced by the Government was to limit the quantity of vaccine expiring. Out of the 391,200 doses of Covishield vaccines received in April 2021, 364, 180 doses remained after the 30 first days which represents 93% of the received doses, to expire in July and August 2021.

**Vaccine hesitancy, and its spillover effect on mass vaccination campaigns:** the COVID-19 context and its rumors, had a negative impact on ongoing mass vaccination campaigns in Cameroon. During the national immunization days for the vaccination campaign against polio, targeting children under 5 years old, but a significant proportion of the population refused to take the oral polio vaccines thinking the government was forcing COVID-19 vaccines on them; Unlike the COVID-19 vaccine, OPV is administered orally, but a certain misunderstanding has sowed confusion in the minds which prevented a successful development of this operation in several regions of the country. The population believed that the National immunization days was a pretext for administering the vaccine against the COVID-19 to the populations and even more so to children.

On Tuesday, April 27, 2021 a contradictory debate was held as part of the activities of the African immunization week on the theme “Overcoming vaccine hesitancy in Cameroon”. This round table brought together eminent personalities, local elected officials, members of the media, bloggers and influencers, the officials of the ministry of Health, the directors of hospitals, the technical and financial partners and the leaders of civil society. It emerged from the round table that vaccine hesitation was accentuated with the arrival of the vaccine against COVID-19 and especially linked to certain peculiarities: the speed of marketing of this vaccine; the diversity of the vaccines and the manufacturing agencies involved; the high political involvement with the COVID-19 vaccine in particular which according to some Cameroonian made it even more suspicious; some of the participants thought that the targets of the vaccines are “guinea pigs for pharmaceutical companies whose mercantilism is very often above the well-being of individuals”.

**Implementation in emergency mode, putting the horse before the cart:** implementation in emergency mode has offered little time for scaling up communication. New vaccine's introduction generally requires significant preparation and structural complexity to ensure optimal vaccine delivery. Thirty days after the introduction of COVID-19 vaccines the risk communication plan was not yet validated. The implementation of interpersonal communication activities, especially in urban health centers, was difficult due to the circulating rumors about the vaccination against COVID-19. The population felt that the Expanded Program on Immunization did not communicate long enough in advance. Some key opinion leaders complained that they did not know how the vaccines looked like, but they were already been administered.

**Cumbersome disbursements procedures:** cumbersome financial and administrative procedures have made it difficult to respond immediately to urgent needs leading to a delay in setting up the technical assistance. The delays in disbursements led to underfunding of preparatory activities which in turn has led to a delay in setting up the minimum conditions for introduction; in addition the small quantity of vaccine allocation that is 600 hundred thousands of doses to be distributed nationally has generated high operational cost. Ensuring long-term financing of operational costs integrating the vaccination against COVID-19 into routine activities while securing long-term funding of operating expenses remained challenging.

**Lessons learned:** despite these challenges, some approaches used to increase vaccine uptake of COVID-19 vaccines could be instructive, and could be applied to many countries around the world, especially low-income countries.

**Political leadership:** the involvement of the Prime Minister, all the Ministers, traditional authorities like Sultans, Lamidos, Governors, heads of hospitals have all received their jab in front of the media and their COVID-19 vaccination was broadcasted live on national television. This gesture has within just two days of the launch created a momentum around and influenced positively those around them to get vaccinated.

**Intensive vaccination campaign brought the vaccines close to the populations and improved uptakes:** the intensive 5-day vaccination campaign started on April 26, 2021 which brought a tenfold increase compare to non-campaign day and improved the overall uptake. During these intensive vaccination campaigns, mobile teams and sites were increased, spaces have been set up to house vaccination points in order to facilitate the population's access to the administration of the vaccine. The mobile strategy is more effective than the fixed strategy to reach the populations. The daily trends analysis showed that during the intensive vaccination campaigns the daily doses administered were high and has started dwindling at the end of the campaign. Achieving high immunization coverage through intensive vaccination campaigns is a best practice that needs to be renewed and scale up. Using influential community members as mobilizers improves the acceptability of people. Awareness by qualified health personnel is an asset for vaccine acceptance, the door-to-door strategy works best in this context, the use of a religious or community leader works

**Invest in early proactive communication:** a month after starting the vaccination communication and demand generation for COVID-19 vaccines has been very slow, centralized and, weak. As such communications must start early to prepare communities to receive the vaccine. The introduction of new vaccines follows stringent protocols with an overdose of communication which has not been the case during the hasty introduction of COVID-19 vaccines. The good intention to avoid delays in the implementation and the start of vaccination to give the protection to those who need them the most may have hurt the communication and maybe the trust. Consumer habits have been fundamentally transformed and are essentially geared towards immediacy and proximity. Local communication needs to start early enough to reach customers and prospects in specific catchment area. It is imperative to make accurate information available to the populations, to build a climate of trust with them. This will make it possible to avoid rumors and to dispel the fear entertained by certain manipulators.

The non-involvement of the heads of health areas reinforces the reluctance and rejection of vaccination in the community and in the health facilities, on the contrary, where there is an involvement of the latter the participation community and vaccination adherence is high. The involvement of traditional, religious and political leaders encourages the support of the populations. Awareness by qualified health personnel is an asset for vaccine acceptance, as non-vaccination of health workers is a form of mistrust that increases vaccine hesitancy. In the northern regions, good acceptance from the populations was observed when the involvement of traditional and religious leaders was effective, however, some targets were difficult to achieve due to the insecurity situation in some regions.

## Discussion

COVID-19 vaccination was introduced in Cameroon on April 12, 2021, for high-risk groups. However, despite the various efforts made at all levels, vaccination coverage is still very low. Despite the goal to vaccinate at least 40% of the total population by December 2022 [3]. If the country does not reach high vaccination levels, the virus is likely to continue circulating in pockets. The national coverage rate for the first dose is 5.4 percent. This coverage is notably lower for women. This vaccine uptake per sex does not reflect the Cameroonian population composed of 48% men and 52% women. Therefore, it is essential to study variations in immunization coverage according to income and geographic location to understand why COVID-19 vaccination coverage is low for women and how gender roles and activities can prevent women's immunization.

Given the newness of the COVID-19 vaccines at that time, AEFI linked to it was not yet fully known. This assessment remains necessary to provide a more complete benefit-risk profile. Unfortunately, limited data was captured on AEFI on the population vaccinated. Secondly, the majority of those vaccinated did not come for their second doses and this made it even more difficult to follow up on AEFI after the first dose. Low rate of use of vaccination services

Vaccine hesitancy coupled with the very close expiry date of the Covishield vaccine put enormous pressure on the national immunization systems. The absence of micro-planning in the health districts has impaired with the involvement of actors at the operational level. Also, the lack of simulation exercises to test coordination particularly regarding the simultaneous deployment of several vaccine products and the measures to be taken in the event of a shortage might have favored the low uptake.

Given the high likelihood of not being able to administer all the doses of vaccines, strategies to increase the uptake and reverse the trends may include increasing the number and distribution of vaccination centers to take vaccines closer to the population. The COVID-19 vaccination in Cameroon was initiated with two types of vaccines: Covishield and Sinopharm. Later on, the country ordered 4 million doses of COVID-19 vaccines of the Janssen via the African Union launched the African Vaccine Acquisition (AVAT) which aims at securing vaccine doses to complement initiatives such as COVAX. By this mechanism 158,400 doses of Johnson & Johnson vaccines were received on July and August 2021 [6]. By September 2021,1.05 million doses have been received and the country has administered 395k doses. The availability of these different types of COVID-19 vaccines represents an opportunity to mitigate the effects of the global pandemic.

## Conclusion

This is the first systematic report that presents the evolution of events following COVID-19 vaccine introduction in Cameroon to the best of our knowledge. It gives an early overview of the challenges, the achievements, and the lessons learned as a contribution to significant discussions on strategies to improve service delivery and vaccine uptake. Notwithstanding, the conclusions of this paper are subject to some limitations because the analysis relied on preliminary and aggregated data and could be subject to delays in reporting. The two main obstacles remained the lack of funding and the weak communication activity due to the slow disbursement of funds. It is urgent to implement effective communication and strong community engagement to make the vaccination against COVID-19 a success in this context.

### What is known about this topic

- COVID-19 is a new disease, and Cameroon's COVID-19 vaccination experience is still unique. As it is the case with other countries, it with was learning by doing as the vaccination of adults is still embryonic in our setting.

### What this study adds

- First national-level study on COVID-19 vaccination since its rollout in April 2021. The study demonstrates the importance of targeted specific communication for a successful rollout of COVID-19 vaccination;
- Timely mobilization of resources is necessary to ensure adequate uptake and vaccines consumption;
- It might be difficult to achieve success when the basics are not covered.
